# Association of low-level lead exposure with all-cause and cardiovascular disease mortality in US adults with hypertension: evidence from the National Health and Nutrition Examination Survey 2003–2010

**DOI:** 10.1186/s13690-023-01148-6

**Published:** 2023-08-14

**Authors:** Lili Wang, Chaofan Wang, Tao Liu, Haochen Xuan, Xiaoqun Li, Xiangxiang Shi, Feng Dai, Junhong Chen, Dongye Li, Tongda Xu

**Affiliations:** 1 Department of Cardiology, The Affiliated Hospital of Xuzhou Medical University, Xuzhou, 221000 China; 2 Department of Cardiology, Jinshan Branch of Shanghai Sixth People’s Hospital, Shanghai, 201500 China; 3 Department of General Practice, The Affiliated Hospital of Xuzhou Medical University, Xuzhou, 221000 China

**Keywords:** Hypertension, Blood lead, All-cause mortality, Cardiovascular disease mortality, Cohort study

## Abstract

**Background:**

To explore the association of low-level lead exposure with all-cause mortality and cardiovascular disease (CVD) mortality among hypertensive patients.

**Methods:**

This cohort study enrolled 6453 adults with hypertension from the National Health and Nutrition Examination Survey 2003–2010 and followed mortality information through December 31, 2019. The baseline population were divided into four groups based on quartiles of blood lead levels (Q1: < 1.2 μg/dL, Q2: 1.2–1.6 μg/dL, Q3: 1.7–2.4 μg/dL, Q4: 2.5–4.9 μg/dL). The correlation of blood lead levels to mortality was investigated by Kaplan–Meier survival curves, restricted cubic spline (RCS), proportional hazard regression model, and subgroup analysis.

**Results:**

During a median follow-up period of 136 (interquartile range 113, 164) months, a total of 1943 (30.1%) deaths were documented, among which 553 (28.5%) were due to CVD. Blood lead showed a linear dose–response relationship with all-cause and CVD mortality. After adequate adjusting for confounders, the risk of all-cause death rose by 23% for each unit increase in continuous variable blood lead (hazard ratio (HR): 1.23; 95% confidence interval (CI):1.16–1.30). When blood lead was a quartile group variable, participants in the Q 4 group had a 73% higher risk of death than those in the Q 1 group (HR:1.73; 95% CI: 1.43–2.10; *P* for trend < 0.001). The association for CVD mortality was analogous. The concordant results were achieved in the subgroup analysis.

**Conclusion:**

Elevated blood lead levels were strongly associated with an increased all-cause and CVD mortality in adults with hypertension, even at the reference range of blood lead.

**Supplementary Information:**

The online version contains supplementary material available at 10.1186/s13690-023-01148-6.

**Table:** 

Text box 1. Contributions to the literature
**•** The relatively low blood lead concentrations, one factor that is frequently overlooked in increasing the risk of death in American adults.
**•** The study showed that blood lead concentrations below the reference range (< 5 μg/dL) were still strongly associated with all-cause and cardiovascular disease mortality.
**•** These findings could contribute to the development of strategies to reduce mortality, such as strict control of lead in food and the environment.

## Introduction

Lead is a widespread heavy metal with more complex effects on humans than other heavy metals because of its stable and non-degradable nature. Not only does lead play no useful role in the human body, but it also adversely affects multi-system organs, and even induces permanent damage [1, 2]. It has been well established that high levels of lead exposure strongly correlate with an increased prevalence of diseases in the cardiovascular, neurological, respiratory, reproductive and urinary systems, and even mortality in humans [1, 3–5]. The Centers for Disease Control and Prevention (CDC) has proposed that the reference blood lead level (BLL) for adults drops from less than 10 µg/dL to less than 5 µg/dL [6]. However, an increasing number of epidemiological studies have shown that the adverse health effects of lead persist even at low levels of exposure [6–10]. Several cross-sectional studies have shown that the BLL correlated with an increased prevalence of hypertension, both high-level and low-level lead exposure [11, 12]. Lead may exacerbate the damage to blood vessels through mechanisms such as enhancing oxidative stress and stimulating the renin–angiotensin–aldosterone system, with a consequent increased risk of hypertension, cardiovascular disease and kidney disease [13]. In hypertensive people, elevated blood lead can promote atherosclerosis and thrombosis, and even accelerate the occurrence of hypertension complications [14].

Published studies of the relationship between blood lead levels and mortality risk have shown inconsistent results. One study reported a J-shaped association between lead levels and all-cause mortality [15]. However, one study in patients with type 2 diabetes indicated a linear dose–response relationship between blood lead and all-cause and cardiovascular disease (CVD) mortality [16]. In addition, another study did not observe an association between blood lead concentration and CVD mortality [17]. Overall, the relationship between lead levels and mortality remains controversial. Heretofore most of the studies about the relationship between lead exposure and mortality have been based on high levels of lead, while few studies were on low levels and even fewer studies have been conducted in hypertensive populations. The association of low-level lead exposure with mortality in hypertensive patients has not been elucidated. Therefore, the purpose of this project was to elucidate the association between low levels of blood lead with all-cause and CVD mortality in hypertensive patients by analyzing nationally representative sample data conducted in the United States.

## Materials and methods

### Study population

The National Health and Nutrition Examination Survey (NHANES) is a program of studies based on the US population, conducted every two years, using a stratified multistage sampling design that combines interviews, questionnaires, physical examinations, and laboratory data to monitor the health and nutritional status of the U.S. population [18]. All procedures and protocols were approved by the National Center for Health Statistics Research Ethics Review Board, and informed consent was obtained from all participants (Ethical review batch number: Protocol #98–12, Protocol #2005–06, Continuation of Protocol #2005–06).

**Fig. 1:**
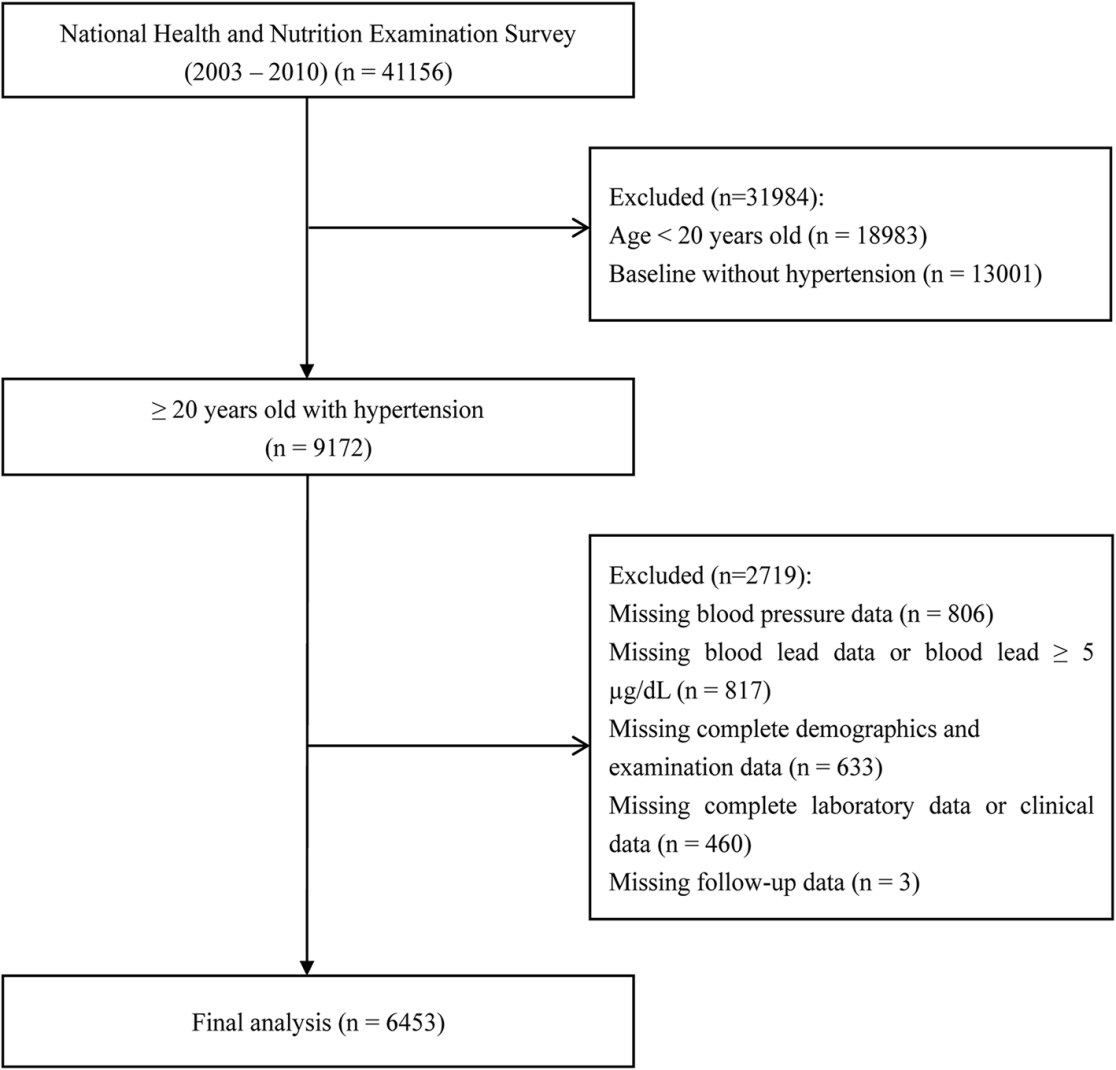
Selection flowchart of study participants from the National Health and Nutrition Examination Survey (NHANES) 2003–2010

A total of 41,156 participants registered for the NHANES from 2003 to 2010 (four two-year cycles). Those aged < 20 years old (*n* = 18,983), no hypertension at baseline (*n* = 13,001), no blood pressure data (*n* = 806), no blood lead data or blood lead ≥ 5 μg/dL (*n* = 817), non-complete demographic and examination data (*n* = 633), non-complete laboratory and clinical data (*n* = 460), and no follow-up data (*n* = 3) were excluded, ultimately 6453 participants were enrolled in the study (Fig. 1).

### Assessment of blood lead

Whole blood samples were processed, stored and transported to the CDC for standardized analysis and quality control. Blood lead concentrations were determined using inductively coupled plasma mass spectrometry [19]. The lower detection limit for blood lead was 0.25 μg/dL (2003–2010), and in the case of tests below the limit of detection, the value of blood lead was determined as the detection limit divided by the square root of two. More detailed descriptions of sample processing are available from the Laboratory Methods section of the web page.

### Covariates

Covariate information was extracted from four separate sections: Demographic data, Examination data, Laboratory data, and Questionnaire data. Demographic information such as sex (Male and Female), age, race (Mexican American, Other Hispanic, Non-Hispanic White, Non-Hispanic Black, and Other Race), poverty income ratio (PIR), and education level (High school or less, College or above) was gained through interviews and questionnaires. Blood pressure (BP) determinations (systolic and diastolic) and body measurements are taken in the Mobile Examination Center. Body mass index (BMI) was obtained after dividing weight (in kilograms) by the square of height (in meters). According to the BMI value, participants were classified as normal weight (18.5–24.9 kg/m^2^), overweight (25.0–29.9 kg/m^2^), and obesity (30.0 kg/m^2^ and higher). Hypertension was defined as systolic BP ≥ 140 mm Hg, diastolic BP ≥ 90 mm Hg, using medications to lower BP, or the self-reported history of hypertension. Diabetes was defined as the self-reported history of diabetes, fasting plasma glucose ≥ 126 mg/dL, hemoglobin A1c ≥ 6.5%, or taking medications to lower blood sugar [20]. Hyperlipidemia was defined by LDL-cholesterol (≥ 130 mg/dL), HDL-cholesterol (male < 40 mg/dL or female < 50 mg/dL), triglycerides (≥ 200 mg/dL), total cholesterol (≥ 150 mg/dL), or taking medications to lower blood lipids [21]. CVD consist of congestive heart failure, coronary heart disease, and stroke. Medication use was obtained from the questionnaire, which included antihypertensive drugs, antidiabetic drugs, antihyperlipidemic drugs, and antiplatelet drugs. estimated Glomerular filtration Rate (eGFR) was calculated from the epidemiological equation of chronic kidney disease [22]. Smokers were defined as those who have smoked at least 100 cigarettes in their lifetime and are still smoking. Drinkers were those who had at least 12 drinks of any type of alcoholic beverage in any one year (1 drink was equal to 12 oz of beer, 4 oz of wine, or one ounce of liquor). More details of covariates are available by visiting https://www.cdc.gov/nchs/nhanes/index.htm.

### Outcomes and follow-up

The outcome events were all-cause death and CVD death. The follow-up period was defined as from the date of the interview to the date of death (for deceased persons) or to the end of the follow-up, i.e., 31 December 2019 (for persons still alive at the end of the study period). Participants who are assumed alive are assigned the number of person-months at the end of the mortality period, December 31, 2019. All-cause mortality means "mortality from all causes of death". The underlying cause of death code is coded according to the 10th revision of the International Statistical Classification of Diseases, Injuries, and Causes of Death (ICD-10). CVD deaths were coded I00-I09, I11, I13, I20-I51, and I60-I69. Public-use Linked Mortality Files are available from the National Center for Health Statistics (NCHS), and survey data from which have been linked to the National Death Index data containing information on mortality status, date of death, and cause of death.

### Statistical analysis

Based on a complex sampling design, sample weights, and stratification were taken into account during statistical analysis. Subjects were classified into quartiles based on BLLs (Q1: < 1.2 μg/dL, Q2: 1.2–1.6 μg/dL, Q3: 1.7–2.4 μg/dL, Q4: 2.5–4.9 μg/dL). Continuous variables were represented as Means ± Standard Deviation or Median (interquartile), with categorical variables in frequency (percentage). The chi-square tests were performed for categorical variables, while Kruskal–Wallis tests were conducted for continuous variables to compare the baseline characteristics of patients in the blood lead quartile subgroup. Schoenfeld residuals showed that all covariables in this study were consistent with the proportional risk hypothesis (all *P* > 0.05). Cox proportional risk regression models were applied to assess the relationship between BLLs and all-cause mortality. Three models were constructed to gradually adjust for confounding factors: Model 1: Unadjusted model; Model 2: Adjusting for sex, age and race; Model 3: Confounders such as BMI, education level, systolic BP, diastolic BP, smoker, drinker, eGFR, diabetes, CVD, hyperlipidemia, antidiabetic drugs, antihypertensive drugs, antihyperlipidemic drugs, and antiplatelet drugs were further adjusted on the basis of Model 2. The adjusted covariates were based on confounding factors mentioned in previous literature, as well as variables that were meaningful for the univariate analysis. The Backward stepwise regression was used for model 3 to avoid multicollinearity. Kaplan–Meier survival curve and Log-rank test were used to compare the inter-group differences in survival between four groups. In addition, the dose–response relationship between blood lead with all-cause and CVD mortality was illustrated by the restricted cubic spline (RCS) plot. In the next step, we performed subgroup analysis and multiplicative interaction analysis stratified by age, sex, smoker, drinker, diabetes, CVD, hyperlipidemia, antidiabetic drugs, antihypertensive drugs, antihyperlipidemic drugs, and antiplatelet drugs. Data analysis was completed by Stata software (version 17.0) and R software (version 4.1.3), and a two-sided *P* value of 5% significance level was considered statistically significant.

## Results

### Baseline parameters

A total of 6453 hypertensive participants who were ≥ 20 years old with blood lead < 5 μg/dL were included in this study, with an average age of 60.1 ± 15.3 years, of whom 3222 (49.9%) were male. Participants were divided into four groups according to blood lead quartiles, and baseline characteristics are shown in Table 1. Participants in the Q4 group were more likely to be older, male, and non-Hispanic compared to participants in the Q1 group. These individuals with higher BLLs had lower BMI, PIR, and education levels, and a majority reported smoking and drinking history. In addition, patients with higher BLLs were more likely to have CVD and take anti-hyperlipidemia and antiplatelet medications.

**Table 1:** Weighted characteristics of the study population by blood lead quartiles, NHANES 2003–2010

	**Blood lead (μg/dL)**					
	**Total**	**Quartile 1**	**Quartile 2**	**Quartile 3**	**Quartile 4**	***P***
Patients, n	6453	1624	1603	1615	1611	
Age, n (%)						< 0.001
60	2733 (42.4)	961 (72.4)	713 (58.3)	591 (48.2)	468 (40.6)	
≥ 60	3720 (57.6)	663 (27.6)	890 (41.7)	1024 (51.8)	1143 (59.4)	
Sex, n (%)						< 0.001
Male	3222 (49.9)	573 (38.3)	776 (50.3)	868 (52.5)	1005 (60.4)	
Female	3231 (50.1)	1051 (61.7)	827 (49.7)	747 (47.5)	606 (39.6)	
Body massing index, n (%)						< 0.001
Normal weight (< 25.0 kg/m^2^)	1262 (19.6)	225 (15.0)	295 (18.7)	335 (22.1)	407 (25.9)	
Overweight (25.0 – 29.9 kg/m^2^)	2183 (33.8)	440 (26.6)	527 (34.1)	578 (35.2)	638 (39.1)	
Obesity (≥ 30.0 kg/m^2^)	3008 (46.6)	959 (58.4)	781 (47.2)	702 (42.8)	566 (35.0)	
Systolic blood pressure, mmHg	132.0(119.0, 144.0)	127.0(117.0, 141.0)	131.0(119.0, 144.0)	133.0(121.0, 145.0)	136.0(122.0, 148.0)	< 0.001
Diastolic blood pressure, mmHg	74.0(65.0, 83.0)	74.0(66.0, 83.0)	74.0(65.0, 83.0)	74.0(65.0, 83.0	73.0(64.0, 83.0)	0.087
Lead, μg/dL	1.6(1.1, 2.3)	0.9(0.7, 1.0)	1.4(1.3, 1.5)	2.0(1.8, 2.2)	3.1(2.7, 3.7)	< 0.001
Race, n (%)						0.020
Mexican American	980 (15.2)	279 (5.8)	244 (5.0)	233 (5.0)	224 (5.0)	
Other Hispanic	365 (5.7)	138 (4.1)	93 (2.6)	76 (2.8)	58 (1.9)	
Non-Hispanic White	3496 (54.2)	852 (75.0)	890 (77.0)	897 (76.5)	857 (73.2)	
Non-Hispanic Black	1383 (21.4)	296 (10.7)	320 (10.5)	352 (11.5)	415 (14.6)	
Other Race	229 (3.5)	59 (4.4)	56 (4.8)	57 (4.3)	57 (5.3)	
Poverty income ratio, n (%)						< 0.001
< 1.3	1866 (28.9)	472 (19.1)	415 (15.5)	451 (18.7)	528 (22.8)	
1.3—3.5	2601 (40.3)	632 (36.9)	638 (38.6)	649 (37.8)	682 (42.2)	
> 3.5	1986 (30.8)	520 (44.0)	550 (45.9)	515 (43.5)	401 (35.0)	
Education Level, n (%)						< 0.001
High school or less	3626 (56.2)	827 (41.5)	855 (44.0)	930 (50.3)	1014 (55.4)	
College or above	2827 (43.8)	797 (58.5)	748 (56.0)	685 (49.7)	597 (44.6)	
Smoker, n (%)						< 0.001
No	5295 (82.1)	1435 (87.4)	1368 (84.8)	1298 (79.5)	1194 (71.60)	
Yes	1158 (17.9)	189 (12.6)	235 (15.2)	317 (20.5)	417 (28.40)	
Drinker, n (%)						0.003
No	2711 (42.0)	750 (39.2)	692 (35.8)	626 (32.1)	643 (33.7)	
Yes	3742 (58.0)	874 (60.8)	911 (64.2)	989 (67.9)	968 (66.3)	
Estimated glomerular filtration rate,ml/min/1.73 m ^2^						< 0.001
< 90	3924 (60.8)	740 (41.9)	977 (58.9)	1064 (63.1)	1143 (69.3)	
≥ 90	2529 (39.2)	884 (58.1)	626 (41.1)	551 (36.9)	468 (30.7)	
Diabetes, n (%)						< 0.001
No	4707 (72.9)	1086 (73.7)	1158 (79.3)	1224 (81.3)	1239 (81.5)	
Yes	1746 (27.1)	538 (26.3)	445 (20.7)	391 (18.7)	372 (18.5)	
Hyperlipidemia, n (%)						0.180
No	1056 (16.36)	269 (17.35)	242 (15.35)	251 (14.49)	294 (17.48)	
Yes	5397 (83.64)	1355 (82.65)	1361 (84.65)	1364 (85.51)	1317 (82.52)	
Cardiovascular diseases, n (%)						< 0.001
No	5113 (79.2)	1385 (88.7)	1294 (85.0)	1243 (79.5)	1191 (76.3)	
Yes	1340 (20.8)	239 (11.3)	309 (15.0)	372 (20.5)	420 (23.7)	
Cancer, n (%)						0.130
No	5541 (85.9)	1425 (87.9)	1372 (85.8)	1384 (86.1)	1360 (84.3)	
Yes	912 (14.1)	199 (12.1)	231 (14.2)	231 (13.9)	251 (15.7)	
Antihypertensive agents, n (%)						0.180
No	5387 (83.5)	1334 (82.3)	1308 (83.1)	1346 (83.3)	1399 (85.8)	
Yes	1066 (16.5)	290 (17.7)	295 (16.9)	269 (16.7)	212 (14.2)	
Antidiabetic agents, n (%)						< 0.001
No	5291 (82.0)	1242 (81.9)	1307 (86.8)	1345 (87.4)	1397 (90.6)	
Yes	1162 (18.0)	382 (18.1)	296 (13.2)	270 (12.6)	214 ( 9.4)	
Antihyperlipidemic agents, n (%)						< 0.001
No	4300 (66.6)	1137 (73.4)	1029 (68.0)	1045 (66.0)	1089 (68.1)	
Yes	2153 (33.4)	487 (26.6)	574 (32.0)	570 (34.0)	522 (31.9)	
Antiplatelet agents, n (%)						< 0.001
No	5987 (92.8)	1529 (96.3)	1497 (95.1)	1482 (92.5)	1479 (93.3)	
Yes	466 (7.2)	95 (3.7)	106 (4.9)	133 (7.5)	132 (6.7)	
All cause mortality, n (%)						< 0.001
No	4510 (69.9)	1331 (86.5)	1184 (79.5)	1106 (74.4)	889 (61.6)	
Yes	1943 (30.1)	293 (13.5)	419 (20.5)	509 (25.6)	722 (38.4)	
Cardiovascular disease mortality, n (%)						< 0.001
No	5900 (91.4)	1551 (96.4)	1489 (94.5)	1466 (92.7)	1394 (88.8)	
Yes	553 (8.6)	73 ( 3.6)	114 ( 5.5)	149 ( 7.3)	217 (11.2)	

Data shown as median (interquartile) or frequency (percentage)

During a median follow-up of 136 (Interquartile range: 113, 164) months, 1943 (30.1%) participants died, of which 553 were attributed to CVD. Participants in survival group were more likely to be younger, non-Hispanic black, and non-drinkers. They had higher level of BMI, PIR, education, diastolic BP and eGFR, lower level of systolic BP, and lower percentages of comorbidities (diabetes, CVD, and cancer) and medication use (antidiabetic drugs, antihyperlipidemic drugs, and antiplatelet drugs). No significant differences between groups were observed for sex, smoking history, and antihypertensive agents (Table 2).

**Table 2:** Weighted characteristics for the study population between death and survival group, NHANES 2003–2010

	**Survival**	**Total death**		***P***
		**Cardiovascular death**	**Non-cardiovascular death**	
Patients, n	4510	553	1390	
Age, n (%)				< 0.001
60	2472 ( 67.3)	69 ( 17.8)	192 ( 20.7)	
≥ 60	2038 ( 32.7)	484 ( 82.2)	1198 ( 79.3)	
Sex, n (%)				0.680
Male	2181 ( 49.7)	313 ( 50.8)	728 ( 48.6)	
Female	2329 ( 50.3)	240 ( 49.2)	662 ( 51.4)	
Body massing index, n (%)				< 0.001
Normal weight (< 25.0 kg/m^2^)	766 ( 18.1)	130 ( 25.2)	366 ( 26.3)	
Overweight (25.0 – 29.9 kg/m^2^)	1466 ( 32.9)	191 ( 29.9)	526 ( 36.2)	
Obesity (≥ 30.0 kg/m^2^)	2278 ( 49.0)	232 ( 44.9)	498 ( 37.5)	
Systolic blood pressure, mmHg	130.0(119.0, 143.0)	138.0(123.0, 154.0)	137.0(122.0, 151.0)	< 0.001
Diastolic blood pressure, mmHg	76.0(67.0, 84.0)	66.0(58.0, 76.0)	69.0(59.0, 78.0)	< 0.001
Lead, μg/dL	1.5(1.0, 2.1)	2.0(1.4, 2.8)	1.9(1.3, 2.7)	< 0.001
Race, n (%)				< 0.001
Mexican American	766 ( 5.8)	52 ( 2.9)	162 ( 3.4)	
Other Hispanic	282 ( 3.2)	22 ( 1.8)	61 ( 2.1)	
Non-Hispanic White	2224 ( 73.5)	374 ( 82.7)	898 ( 82.0)	
Non-Hispanic Black	1050 ( 12.2)	93 ( 10.0)	240 ( 9.8)	
Other Race	188 ( 5.3)	12 ( 2.6)	29 ( 2.7)	
Poverty income ratio, n (%)				< 0.001
< 1.3	1217 ( 16.8)	187 ( 27.3)	462 ( 25.6)	
1.3–3.5	1705 ( 35.8)	261 ( 49.4)	635 ( 47.6)	
> 3.5	1588 ( 47.4)	105 ( 23.3)	293 ( 27.8)	
Education Level, n (%)				< 0.001
High school or less	2364 ( 43.1)	374 ( 64.4)	888 ( 58.9)	
College or above	2146 ( 56.9)	179 ( 35.6)	502 ( 41.1)	
Smoker, n (%)				0.670
No	3683 ( 81.5)	462 ( 82.9)	1150 ( 80.8)	
Yes	827 ( 18.5)	91 ( 17.1)	240 ( 19.2)	
Drinker, n (%)				< 0.001
No	2873 ( 69.8)	252 ( 47.1)	617 ( 47.7)	
Yes	1637 ( 30.2)	301 ( 52.9)	773 ( 52.3)	
Estimated glomerular filtration rate,ml/min/1.73 m ^2^				< 0.001
< 90	2308 ( 49.91)	476 ( 84.74)	1140 ( 80.11)	
≥ 90	2202 ( 50.09)	77 ( 15.26)	250 ( 19.89)	
Diabetes, n (%)				< 0.001
No	3454 ( 82.2)	356 ( 65.3)	897 ( 67.9)	
Yes	1056 ( 17.8)	197 ( 34.7)	493 ( 32.1)	
Hyperlipidemia, n (%)				0.900
No	740 ( 16.2)	85 ( 15.5)	231 ( 16.4)	
Yes	3770 ( 83.8)	468 ( 84.5)	1159 ( 83.6)	
Cardiovascular diseases, n (%)				< 0.001
No	3888 ( 88.7)	304 ( 54.5)	921 ( 67.4)	
Yes	622 ( 11.3)	249 ( 45.5)	469 ( 32.6)	
Cancer, n (%)				< 0.001
No	4039 ( 89.3)	449 ( 80.5)	1053 ( 74.3)	
Yes	471 ( 10.7)	104 ( 19.5)	337 ( 25.7)	
Antihypertensive agents, n (%)				0.290
No	3760 ( 83.9)	456 ( 80.7)	1171 ( 82.9)	
Yes	750 ( 16.1)	97 ( 19.3)	219 ( 17.1)	
Antidiabetic agents, n (%)				< 0.001
No	3811 ( 88.8)	416 ( 76.1)	1064 ( 79.4)	
Yes	699 ( 11.2)	137 ( 23.9)	326 ( 20.6)	
Antihyperlipidemic agents, n (%)				< 0.001
No	3184 ( 72.5)	308 ( 54.4)	808 ( 59.1)	
Yes	1326 ( 27.5)	245 ( 45.6)	582 ( 40.9)	
Antiplatelet agents, n (%)				< 0.001
No	4307 ( 96.5)	464 ( 85.5)	1216 ( 88.8)	
Yes	203 ( 3.5)	89 ( 14.5)	174 ( 11.2)	

Data shown as median (interquartile) or frequency (percentage)

### Survival curve

**Fig. 2:**
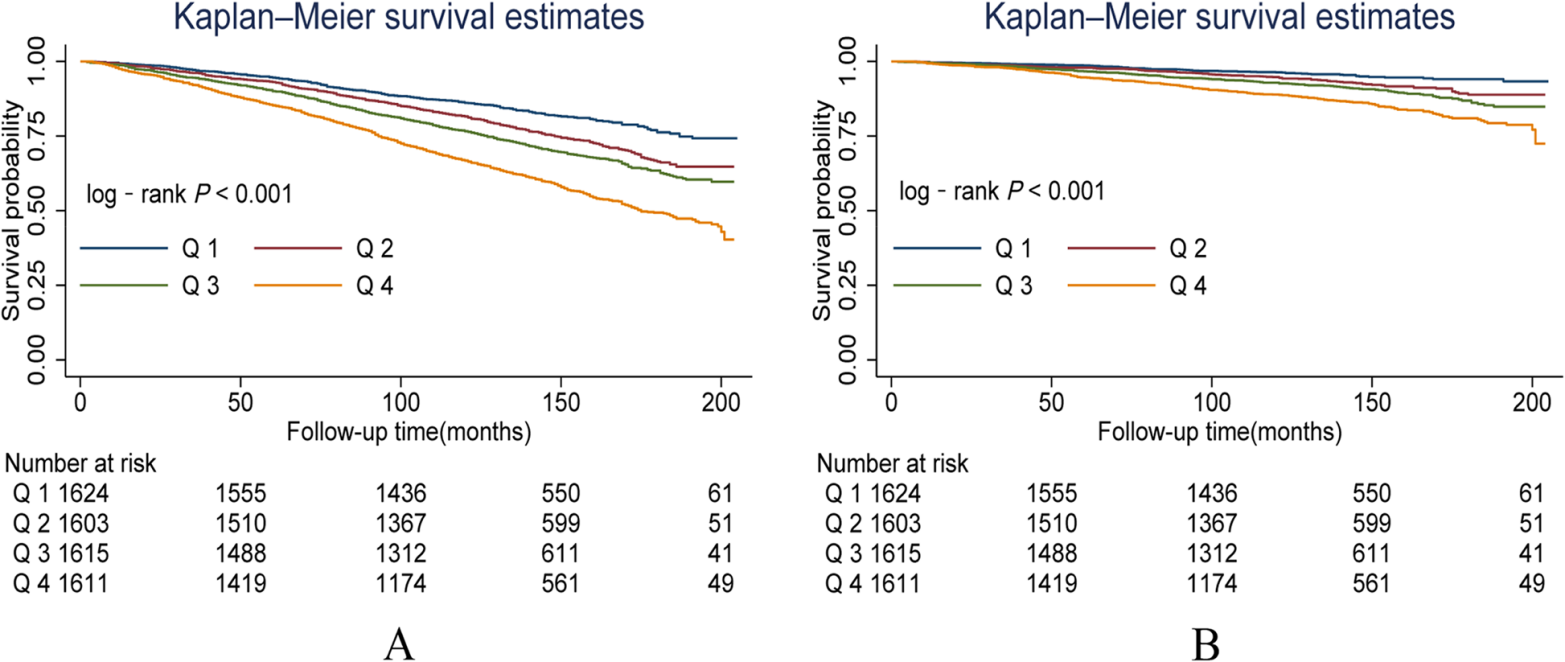
Kaplan–Meier survival curves for all-cause (**A**) and cardiovascular disease mortality (**B**) according to blood lead categories among patients with hypertension (≥ 20 years old) in NHANES 2003—2010

Figure 2 shows the Kaplan–Meier survival curves stratified by BLL. With the increase of BLL, the survival probability of patients gradually decreases. Regarding all-cause mortality (Fig. 2A) and CVD mortality (Fig. 2B), the risk of death was lowest in group Q1 and highest in group Q4, with both log-rank tests demonstrating statistically significant differences between groups (all *P* for log-rank < 0.001).

### Association of blood lead with mortality

As shown in Table 3, proportional risk regression models were performed to assess the relationship between BLLs and with all-cause and CVD mortality. In the fully adjusted model 3, the risk of all-cause mortality increased by 23% for each 1-unit increase in the continuous variable blood lead; When blood lead was quartile grouped, the HR (95% CI) for all-cause mortality were 1.21 ( 0.99–1.48), 1.26 ( 1.05–1.52), and 1.73 ( 1.43–2.10) for the Q2, Q3, and Q4 groups, respectively. In brief, BLLs were positively associated with all-cause mortality in fully adjusted model 3, either as a continuous or categorical variable (all P for trend < 0.05). In a similar way, BLLs were positively associated with CVD mortality when it was considered as a continuous variable (HR: 1.34; 95%CI: 1.21–1.48). When BLL quartiles were treated as a categorical variable, the HR (95% CI) for CVD mortality was 1.22 ( 0.87- 1.72), 1.32 ( 0.95–1.83) and 2.00 ( 1.47–2.73) for groups Q2, Q3, and Q4 respectively, compared with group Q1. The increased risk of CVD death in the Q2 and Q3 group were not significant.

**Table 3:** Multivariate Cox regression analysis for the relationship between blood lead levels with all-cause and cardiovascular disease mortality

	**Model 1 HR(95%CI) *P***	**Model 2 HR(95%CI) *P***	**Model 3 HR(95%CI) *P***
**All-cause mortality**			
Continuous	1.46 ( 1.40, 1.52) < 0.001	1.25 ( 1.19, 1.30) < 0.001	1.23 ( 1.16, 1.30) < 0.001
Lead quartiles			
Q1	1.0	1.0	1.0
Q2	1.51 ( 1.21, 1.89) < 0.001	1.14 ( 0.93, 1.39) 0.22	1.21 ( 0.99, 1.48) 0.06
Q3	1.94 ( 1.61, 2.33) < 0.001	1.21 ( 1.00, 1.46) 0.05	1.26 ( 1.05, 1.52) 0.01
Q4	3.06 ( 2.61, 3.60) < 0.001	1.75 ( 1.47, 2.08) < 0.001	1.73 ( 1.43, 2.10) < 0.001
*p* for trend	< 0.001	< 0.001	< 0.001
**Cardiovascular disease mortality**			
Continuous	1.60 ( 1.48, 1.72) < 0.001	1.35 ( 1.24, 1.48) < 0.001	1.34 ( 1.21, 1.48) < 0.001
Lead quartiles			
Q1	1.0	1.0	1.0
Q2	1.59 ( 1.11, 2.26) 0.01	1.13 ( 0.80, 1.60) 0.47	1.22 ( 0.87, 1.72) 0.24
Q3	2.20 ( 1.64, 2.96) < 0.001	1.26 ( 0.93, 1.72) 0.14	1.32 ( 0.95, 1.83) 0.09
Q4	3.83 ( 2.91, 5.05) < 0.001	2.01 ( 1.51, 2.67) < 0.001	2.00 ( 1.47, 2.73) < 0.001
*p* for trend	< 0.001	< 0.001	0.005

*HR* Hazard Ratio, *CI* Confidence interval

Model 1: Unadjusted model

Model 2: Adjusted for sex, age and race

Model 3: Confounders such as body mass index, education level, poverty income ratio, systolic blood pressure, diastolic blood pressure, smoker, drinker, estimated glomerular filtration rate, diabetes, cardiovascular disease, hyperlipidemia, cancer, antidiabetic drugs, antihypertensive drugs, antihyperlipidemic drugs, and antiplatelet drugs, were further adjusted on the basis of Model 2

**Fig. 3:**
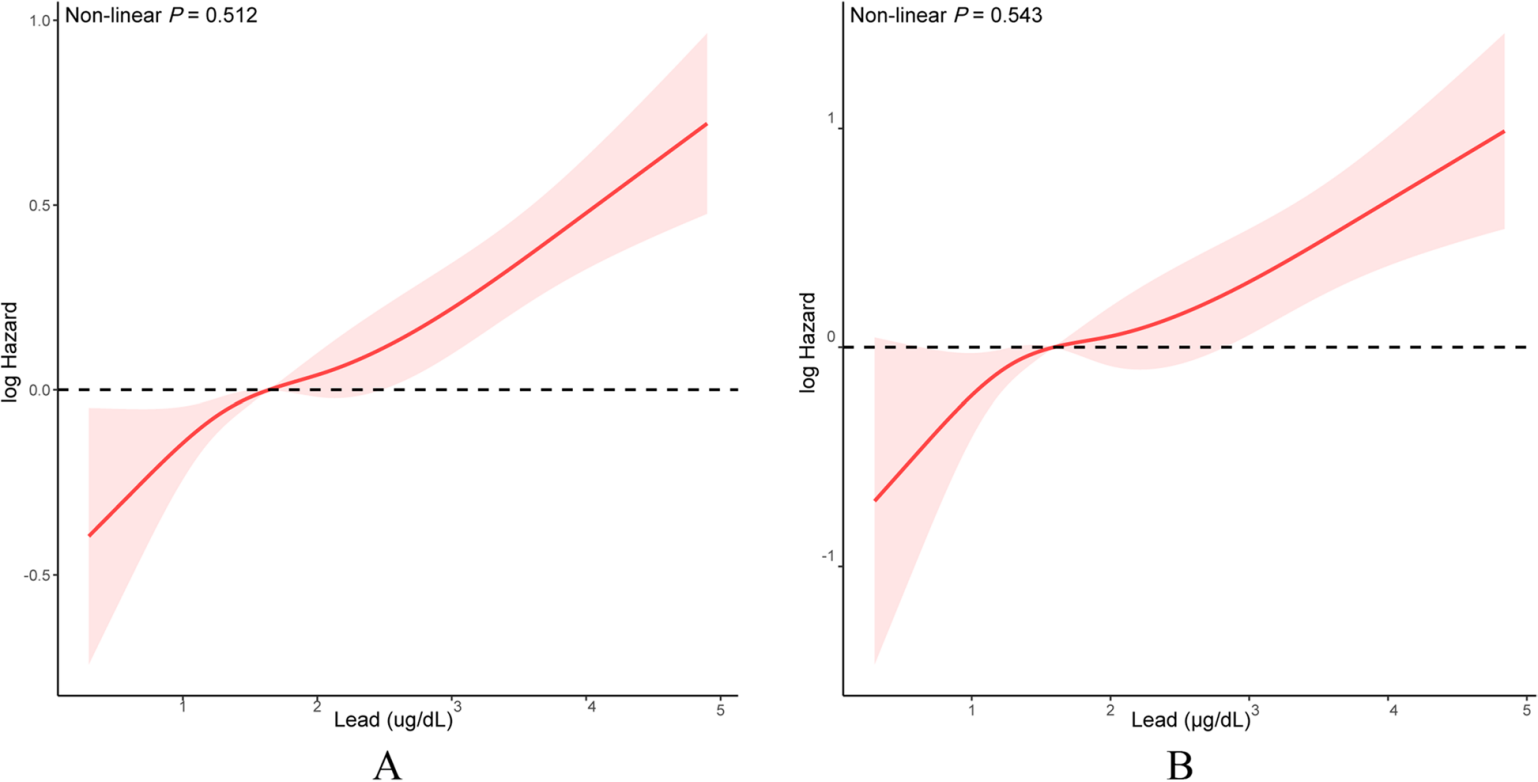
Adjusted cubic spline model of the associations between blood lead with all-cause (**A**) and cardiovascular disease mortality (**B**) among patients with hypertension (≥ 20 years old) in NHANES 2003–2010. Adjusted for age (< 60 or ≥ 60 years), sex (male or female), race (Mexican American, Other Hispanic, Non-Hispanic White, Non-Hispanic Black, Other Race), body mass index (normal weight: 18.5—24.9 kg/m^2^, overweight: 25.0—29.9 kg/m^2^, and obesity: ≥ 30.0 kg/m^2^), baseline systolic blood pressure (contious), baseline diastolic blood pressure (contious), estimated Glomerular filtration rate (< 90 or ≥ 90 ml/min per 1.73m^2^), education level (high school or less, college or above), poverty income ratio (< 1.3, 1.3—3.5, > 3.5), smoker (yes or no), drinker (yes or no), diabetes (yes or no), cardiovascular disease (yes or no), hyperlipidemia (yes or no), antidiabetic drugs (yes or no), antihypertensive drugs (yes or no), antihyperlipidemic drugs (yes or no), and antiplatelet drugs (yes or no)

The dose–response relationship between low levels of blood lead and mortality was explored with restrictive cubic spline plots, and linear relationships (all *P* for non-linear > 0.05) were observed as shown in Fig. 3.

### Subgroup analysis

**Fig. 4:**
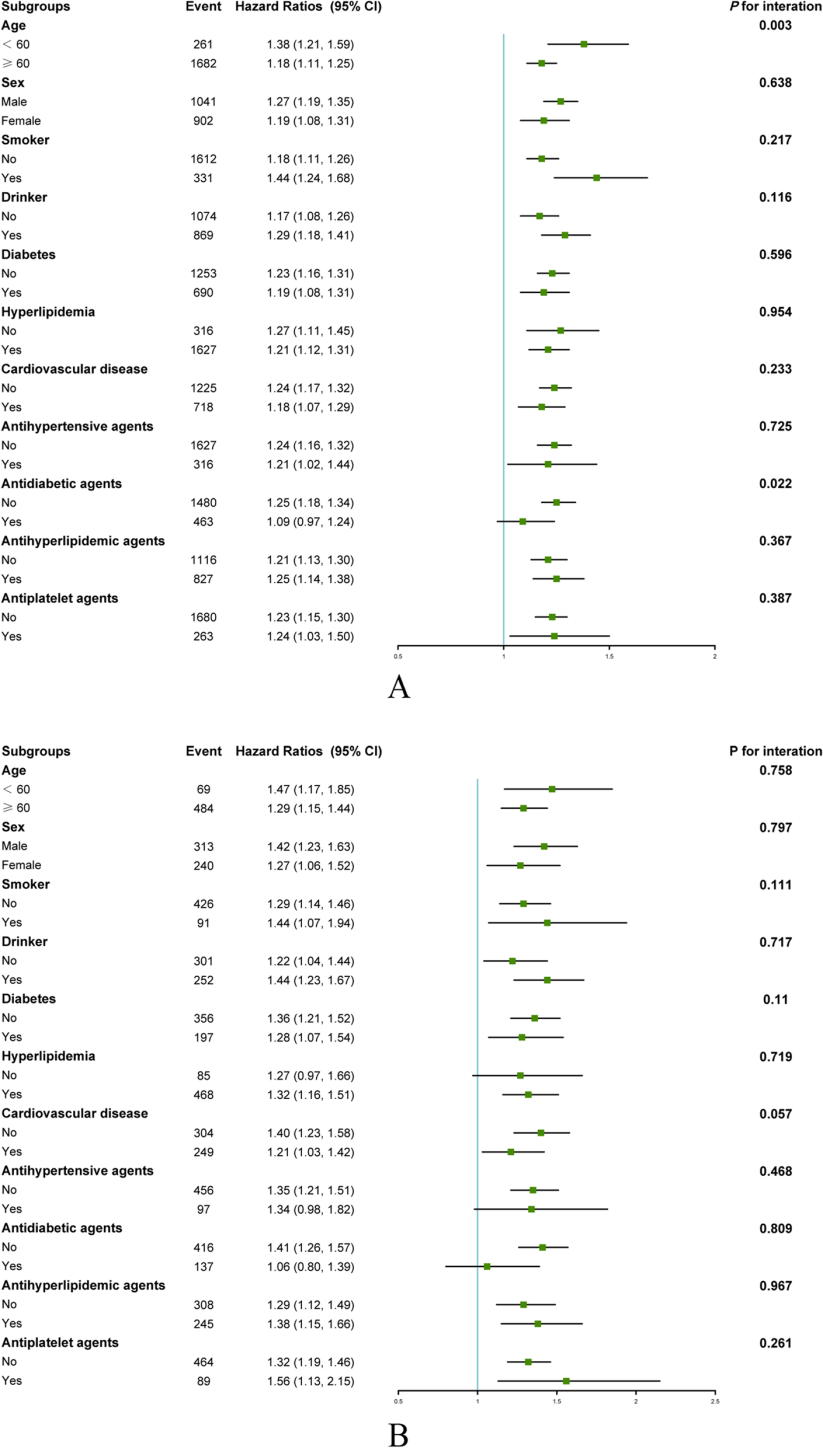
The subgroup analysis of the associations (hazard ratios, 95% CI) between blood lead concentrations and all-cause mortality (**A**) and cardiovascular disease mortality (**B**) among patients with hypertension in NHANES 2003–2010. CI, Confidence interval. HR (95% CI) based on Cox risk proportional regression analysis. Adjusted for age, sex, race, body mass index, baseline systolic blood pressure, baseline diastolic blood pressure, estimated Glomerular filtration rate, education level, poverty income ratio, smoker, drinker, diabetes, cardiovascular disease, hyperlipidemia, cancer, antidiabetic drugs, antihypertensive drugs, antihyperlipidemic drugs, and antiplatelet drugs

In subgroup analyses, we adjusted for age (< 60 or ≥ 60 years), sex (male or female), race (Mexican American, other Hispanic, non-Hispanic white, non-Hispanic black, other race), body mass index (normal weight: 18.5–24.9 kg/m^2^, overweight: 25.0–29.9 kg/m^2^, and obesity: ≥ 30.0 kg/m^2^), education level (high school or less, college or higher), PIR, smoker (yes or no), drinker (yes or no), diabetes (yes or no), CVD (yes or no), hyperlipidemia (yes or no), antihypertensive agents (yes or no), antidiabetic drugs (yes or no), antihyperlipidemic drugs (yes or no), and antiplatelet drugs (yes or no), except as stratified variables for subgroup analysis. As shown in Fig. 4A, the effect of blood lead on all-cause mortality was altered by age and antidiabetic drugs, indicating that lead significantly increased the risk of all-cause mortality in patients who were < 60 years old (HR: 1.38; 95% CI: 1.21, 1.59; *P* for interaction: 0.003) and without a history of glucose-lowering medication (HR: 1.25; 95% CI: 1.18–1.34; *P* for interaction: 0.022). Meanwhile, subgroup analysis was performed to explore the relationship between blood lead and CVD mortality, and there was no interaction between any of the factors and blood lead as shown in Fig. 4B (all *P* for interaction > 0.05). Furthermore, threshold effects analysis were performed by subgroups of age and antidiabetic drugs (Table S1-S2). In addition, RCS curves were applied to show dose–response relationships of lead levels with all-cause mortality in subgroups of age and antidiabetic medications (Figure S1-2).

## Discussion

In this nationally representative longitudinal cohort study, we analyzed data from 6453 adults with hypertension and found that higher blood lead concentrations were associated with a higher prevalence of diabetes and CVD. BLLs were linearly and positively associated with the risk of all-cause and cardiovascular disease mortality even at the reference range of blood lead. In addition, age and hypoglycemic medications interacted significantly with the association between blood lead and risk of all-cause mortality.

Numerous studies based on different populations have shown that lead concentration is an independent risk factor for all-cause and CVD mortality. A longitudinal study of aging males in the Boston area of Massachusetts showed that those with high levels of bone lead (> 35 μg/g) had a 1.52-fold increased risk of all-cause death (HR: 2.52; 95% CI:1.17—5.41) and a 4.63-fold increased risk of cardiovascular death (HR: 5.63; 95% CI:1.73 -18.30) over an average of 8.9 years of follow-up compared with those with low levels of bone lead (< 22 μg/g) [23]. Another cohort study of 18,602 U.S. adults ≥ 40 years old followed for a median of 6.2 years found that log-transformed blood lead was linearly associated with increased CVD mortality [24]. Shi et al. reported a linear relationship between dietary lead intake and all-cause mortality after 9.8 years of follow-up of 2832 Chinese adults [25]. Our findings are partially consistent with the conclusions of the above studies. It can be concluded from the above studies that high levels of lead will increase the risk of death, but there is no unified conclusion on the specific dose–response relationship between lead level and mortality within the previously considered safe range. Lanphear et al. reported that for adults with blood lead concentrations < 5 μg/dL, BLL showed the same increasing trend with all-cause mortality and CVD mortality after 19.3 years of follow-up [26]. Our results were in agreement with their views. Garam Byun et al. investigated the relationship between BLLs (< 10 μg/dL) and mortality in 7308 Korean adults and found that the relationship between BLL and mortality was similar to an inverted U- shape, which was inconsistent with our results [27]. The differences could be explained by differences in study population, follow-up time, and adjustment factors.

It is interesting to note that age and antidiabetic drugs interacted with BLL. In the subgroup analysis, the effect of blood lead on all-cause mortality appeared to be more evident in patients who were not receiving antidiabetic drug protection at baseline. The possible reasons are as follows. Lead can induce oxidative stress and chronic inflammationin the body, leading to cell apoptosis and metabolic disorders of tissues and organs [28]. On the one hand, some antidiabetic drugs, such as metformin and dapagliflozin, can antagonize oxidative stress, diminish inflammatory responses, and improve vascular endothelial function by modulating multiple pathways and play a protective role in the heart, brain, kidney, and other important organs [29, 30]. On the other hand, previous studies have supported that glucose-lowering drugs may reduce the incidence of cancer and associated mortality, which was one of the leading causes of death in hypertensive patients in this study [31, 32]. In addition, the development and severity of hypertension can be slowed by diabetes medication in several ways that have been confirmed by previous studies [33, 34]. As mentioned above, the risk of death was relatively lower in patients using antidiabetic drugs than those not. Hypoglycemic drugs can not only control blood sugar, but also improve complications. Another finding of the subgroup analysis was that the effect of BLL on all-cause mortality was enhanced at age < 60 years. The result is consistent with previous researches. One study reported that the effect of lead on all-cause and on CVD mortality was greater in people younger than 50 years than in older adults [35]. Liu et al. found that the risk of all-cause mortality was significantly higher in patients < 60 years than in patients ≥ 60 years (HR 3.01,95% CI 2.02–4.47 vs 1.31,1.04–1.65), but the interaction was not significant [16]. There is still no exact mechanism to explain this result.

Potential mechanisms of the relationship between lead exposure and mortality in hypertensive patients are as follows. Both animal and human studies have demonstrated that lead exposure can lead to hypertension by increasing renin–angiotensin–aldosterone system reactivity, reducing prostacycline production, and enhancing oxidative stress [1, 9, 36–38]. The antioxidant capacity of hypertensive patients is decreased, and the level of oxidants is increased, which further accelerates the oxidative stress response and eventually leads to mitochondrial damage and cell apoptosis [39, 40]. In addition, NHANES surveys reported that low-level lead exposure was positively correlated with the risk of dyslipidemia (TC, LDL-C, non-HDL-C) in adults, which can lead to endothelial dysfunction and atherosclerosis [41, 42]. In addition, the cardiotoxicity of lead is also reflected in affecting myocardial contractility, damaging the regulation of cardiac excitability and changing the activity of autonomic nervous system. More research is needed to clarify the underlying mechanism in the future.

## Strengths and limitations

The strengths of this study include prospective design, large sample size, and the nationally representative sample of adults with hypertension in the United States. In addition, the data were collected and analyzed in strict compliance with the NHANES protocol, which makes our results more reliable. However, there are some limitations. First of all, the exclusion of patients with missing information on inclusion indicators may lead to selection bias. Secondly, we only analyzed the BLL at the baseline, which may change dynamically over time. Thirdly, residual confounders have not been eliminated, although a large number of potential confounders have been adjusted.

## Conclusion

We found that for hypertensive patients, higher BLLs were associated with an increased risk of all-cause and CVD mortality in linear dose–response forms, even at the reference range. It remains vital for adults to reduce or eliminate lead exposure.

### Supplementary Information

**Additional file 1: Supplementary Table 1.** Thresholdeffect analysis of blood lead on all-cause mortality modified by age. **SupplementaryTable 2.** Threshold effectanalysis of blood lead on all-cause mortality modified by antidiabetic drugs. **SupplementaryFigure 1.** Adjustedcubic spline model of the association between hazard ratio of all-causemortality and blood lead levels in participants < 60 years old (*P* fornon-linear = 0.661) and ≥ 60 years old (*P* fornon-linear = 0.193). **Supplementary Figure 2.** Adjustedcubic spline model of the association between hazard ratio of all-causemortality and blood lead levels in participants without baseline antidiabeticdrugs (*P* for non-linear = 0.902) and with baseline antidiabetic drugs(*P* for non-linear = 0.196)

## Data Availability

The data of this study are publicly available on the NHANES website.

## Abbreviations

BLL: Blood lead level
BMI: Body mass index
BP: Blood pressure
CDC: Centers for Disease Control and Prevention
CI: Confidence interval
CVD: Cardiovascular disease
eGFR: Estimated Glomerular filtration Rate
HR: Hazard ratio
NCHS: National Center for Health Statistics
NHANES: National Health and Nutrition Examination Survey
PIR: Poverty income ratio
RCS: Restricted cubic spline

## References

[1] Boskabady M, Marefati N, Farkhondeh T, Shakeri F, Farshbaf A, Boskabady MH (2018). The effect of environmental lead exposure on human health and the contribution of inflammatory mechanisms, a review. Environ Int.

[2] Mitra P, Sharma S, Purohit P, Sharma P (2017). Clinical and molecular aspects of lead toxicity: an update. Crit Rev Clin Lab Sci.

[3] Staessen JA, Thijs L, Yang WY, Yu CG, Wei FF, Roels HA (2020). Interpretation of population health metrics: environmental lead exposure as exemplary case. Hypertension.

[4] Wei W, Wu X, Bai Y, Li G, Feng Y, Meng H (2020). Lead exposure and its interactions with oxidative stress polymorphisms on lung function impairment: Results from a longitudinal population-based study. Environ Res.

[5] Yao X, Steven XuX, Yang Y, Zhu Z, Zhu Z, Tao F (2021). Stratification of population in NHANES 2009–2014 based on exposure pattern of lead, cadmium, mercury, and arsenic and their association with cardiovascular, renal and respiratory outcomes. Environ Int.

[6] Landrigan PJ, Bellinger D (2021). It’s time to end lead poisoning in the United States. JAMA Pediatr.

[7] Rocha A, Trujillo KA (2019). Neurotoxicity of low-level lead exposure: History, mechanisms of action, and behavioral effects in humans and preclinical models. Neurotoxicology.

[8] Ren J, Cui J, Chen Q, Zhou N, Zhou Z, Zhang GH (2020). Low-level lead exposure is associated with aberrant sperm quality and reproductive hormone levels in Chinese male individuals: Results from the MARHCS study low-level lead exposure is associated with aberrant sperm quality. Chemosphere.

[9] Javorac D, Tatović S, Anđelković M, Repić A, Baralić K, Djordjevic AB (2022). Low-lead doses induce oxidative damage in cardiac tissue: Subacute toxicity study in Wistar rats and Benchmark dose modelling. Food Chem Toxicol.

[10] Liu J, Portnoy J, Um P, Cui N, Rudo-Hutt A, Yan C (2021). Blood lead and mercury levels are associated with low resting heart rate in community adolescent boys. Int J Hyg Environ Health.

[11] Almeida Lopes A, Silbergeld EK, Navas-Acien A, Zamoiski R, Martins A, Camargo A (2017). Association between blood lead and blood pressure: a population-based study in Brazilian adults. Environ Health.

[12] Navas-Acien A, Schwartz BS, Rothenberg SJ, Hu H, Silbergeld EK, Guallar E (2008). Bone lead levels and blood pressure endpoints: a meta-analysis. Epidemiology.

[13] Aune D, Huang W, Nie J, Wang Y (2021). Hypertension and the risk of all-cause and cause-specific mortality: an outcome-wide association study of 67 causes of death in the National Health Interview Survey. Biomed Res Int.

[14] Alghasham AA, Meki AR, Ismail HA (2011). Association of blood lead level with elevated blood pressure in hypertensive patients. Int J Health Sci (Qassim).

[15] Schober SE, Mirel LB, Graubard BI, Brody DJ, Flegal KM (2006). Blood lead levels and death from all causes, cardiovascular disease, and cancer: results from the NHANES III mortality study. Environ Health Perspect.

[16] Zhu K, Zhang Y, Lu Q, Geng T, Li R, Wan Z (2022). Associations of exposure to lead and cadmium with risk of all-cause and cardiovascular disease mortality among patients with type 2 diabetes. Environ Sci Pollut Res Int.

[17] Møller L, Kristensen TS (1992). Blood lead as a cardiovascular risk factor. Am J Epidemiol.

[18] Ezzati TM, Massey JT, Waksberg J, Chu A, Maurer KR. Sample design: Third National Health and Nutrition Examination Survey. Vital Health Stat 2. 1992;(113):1-35. 1413563

[19] Laboratory Procedures Used for the Third National Health and Nutrition Examination Survey (NHANES III) https://www.cdc.gov/nchs/data/nhanes/nhanes3/cdrom/nchs/manuals/labman.pdf.

[20] 2. Classification and Diagnosis of Diabetes: Standards of Medical Care in Diabetes-2020. Diabetes Care. 2020;43(Suppl 1):S14–14S31. 10.2337/dc20-S002. 10.2337/dc20-S002 31862745

[21] Third Report of the National Cholesterol Education Program (NCEP) Expert Panel on Detection, Evaluation, and Treatment of High Blood Cholesterol in Adults (Adult Treatment Panel III) final report. Circulation. 2002;106(25):3143–421. 10.1001/jama.285.19.2486. 12485966

[22] Coresh J, Astor BC, McQuillan G, Kusek J, Greene T, Van Lente F (2002). Calibration and random variation of the serum creatinine assay as critical elements of using equations to estimate glomerular filtration rate. Am J Kidney Dis.

[23] Weisskopf MG, Jain N, Nie H, Sparrow D, Vokonas P, Schwartz J (2009). A prospective study of bone lead concentration and death from all causes, cardiovascular diseases, and cancer in the Department of Veterans Affairs Normative Aging Study. Circulation.

[24] Aoki Y, Brody DJ, Flegal KM, Fakhouri T, Axelrad DA, Parker JD (2016). Blood lead and other metal biomarkers as risk factors for cardiovascular disease mortality. Medicine (Baltimore).

[25] Shi Z, Zhen S, Orsini N, Zhou Y, Zhou Y, Liu J (2017). Association between dietary lead intake and 10-year mortality among Chinese adults. Environ Sci Pollut Res Int.

[26] Lustberg M, Silbergeld E (2002). Blood lead levels and mortality. Arch Intern Med.

[27] Byun G, Kim S, Kim SY, et al. Blood Lead Concentrations and Mortality in Korean Adults: the Korea National Health and Nutrition Examination Survey with Mortality Follow-Up. Int J Environ Res Public Health. 2020;17(18):1–12. 10.3390/ijerph17186898 PMC7557382 32967243

[28] Matović V, Buha A, Ðukić-Ćosić D, Bulat Z (2015). Insight into the oxidative stress induced by lead and/or cadmium in blood, liver and kidneys. Food Chem Toxicol.

[29] Wang G, Wang Y, Yang Q, Xu C, Zheng Y, Wang L (2022). Metformin prevents methylglyoxal-induced apoptosis by suppressing oxidative stress in vitro and in vivo. Cell Death Dis.

[30] Hsieh PL, Chu PM, Cheng HC, et al. Dapagliflozin Mitigates Doxorubicin-Caused Myocardium Damage by Regulating AKT-Mediated Oxidative Stress, Cardiac Remodeling, and Inflammation. Int J Mol Sci. 2022;23(17):1–14. 10.3390/ijms231710146 PMC9456438 36077544

[31] Stevenson-Hoare J, Leonenko G, Escott-Price V (2023). Comparison of long-term effects of metformin on longevity between people with type 2 diabetes and matched non-diabetic controls. BMC Public Health.

[32] Guo M, Shang X, Guo D (2022). Metformin use and mortality in women with ovarian cancer: an updated meta-analysis. Int J Clin Pract.

[33] Kravtsova O, Bohovyk R, Levchenko V, Palygin O, Klemens CA, Rieg T, et al. SGLT2 inhibition effect on salt-induced hypertension, RAAS, and Na(+) transport in Dahl SS rats. Am J Physiol Renal Physiol. 2022;322(6):F692-692F707. 10.1152/ajprenal.00053.2022 PMC9142161 35466690

[34] Zilov AV, Abdelaziz SI, AlShammary A, Al Zahrani A, Amir A, Assaad Khalil SH (2019). Mechanisms of action of metformin with special reference to cardiovascular protection. Diabetes Metab Res Rev.

[35] Lanphear BP, Rauch S, Auinger P, Allen RW, Hornung RW. Low-level lead exposure and mortality in US adults: a population-based cohort study. Lancet Public Health. 2018;3(4):e177–177e184. 10.1016/S2468-2667(18)30025-2 29544878

[36] Simões MR, Azevedo BF, Alonso MJ, Salaices M, Vassallo DV (2020). Chronic low-level lead exposure increases mesenteric vascular reactivity: role of cyclooxygenase-2-derived prostanoids. Front Physiol.

[37] Simões MR, Ribeiro Júnior RF, Vescovi MV, de Jesus HC, Padilha AS, Stefanon I (2011). Acute lead exposure increases arterial pressure: role of the renin-angiotensin system. PLoS ONE.

[38] Dobrakowski M, Pawlas N, Kasperczyk A, Kozłowska A, Olewińska E, Machoń-Grecka A (2017). Oxidative DNA damage and oxidative stress in lead-exposed workers. Hum Exp Toxicol.

[39] Franco C, Sciatti E, Favero G, Bonomini F, Vizzardi E, Rezzani R. Essential Hypertension and Oxidative Stress: Novel Future Perspectives. Int J Mol Sci. 2022;23(22):1–17. 10.3390/ijms232214489 PMC9692622 36430967

[40] Forman HJ, Zhang H (2021). Targeting oxidative stress in disease: promise and limitations of antioxidant therapy. Nat Rev Drug Discov.

[41] Xu H, Mao Y, Xu B, Hu Y (2021). Low-level environmental lead and cadmium exposures and dyslipidemia in adults: findings from the NHANES 2005–2016. J Trace Elem Med Biol.

[42] Lind L, Araujo JA, Barchowsky A, Belcher S, Berridge BR, Chiamvimonvat N (2021). Key characteristics of cardiovascular toxicants. Environ Health Perspect.

