# Supplementary figures and images for: Association of low-level lead exposure with all-cause and cardiovascular disease mortality in US adults with hypertension: evidence from the National Health and Nutrition Examination Survey 2003–2010
Source: Arch Public Health. 2023 Aug 14;81:146. doi: 10.1186/s13690-023-01148-6 (PMC10424362; doi:10.1186/s13690-023-01148-6)

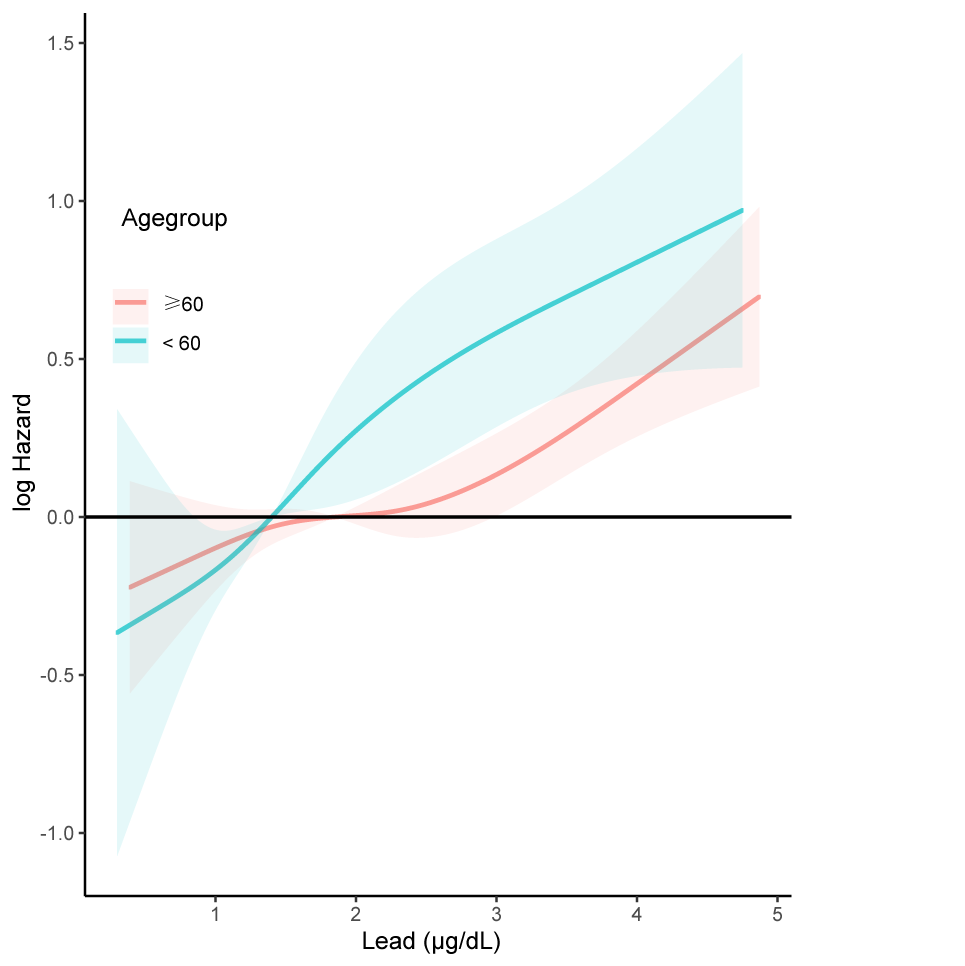

Supplement: Supplementary file 1 — Additional file 1: Supplementary Table 1. Thresholdeffect analysis of blood lead on all-cause mortality modified by age. SupplementaryTable 2. Threshold effectanalysis of blood lead on all-cause mortality modified by antidiabetic drugs. SupplementaryFigure 1. Adjustedcubic spline model of the association between hazard ratio of all-causemortality and blood lead levels in participants < 60 years old (P fornon-linear = 0.661) and ≥ 60 years old (P fornon-linear = 0.193). Supplementary Figure 2. Adjustedcubic spline model of the association between hazard ratio of all-causemortality and blood lead levels in participants without baseline antidiabeticdrugs (P for non-linear = 0.902) and with baseline antidiabetic drugs(P for non-linear = 0.196) [file 13690_2023_1148_MOESM1_ESM.zip › supplement_Figure1.tif]

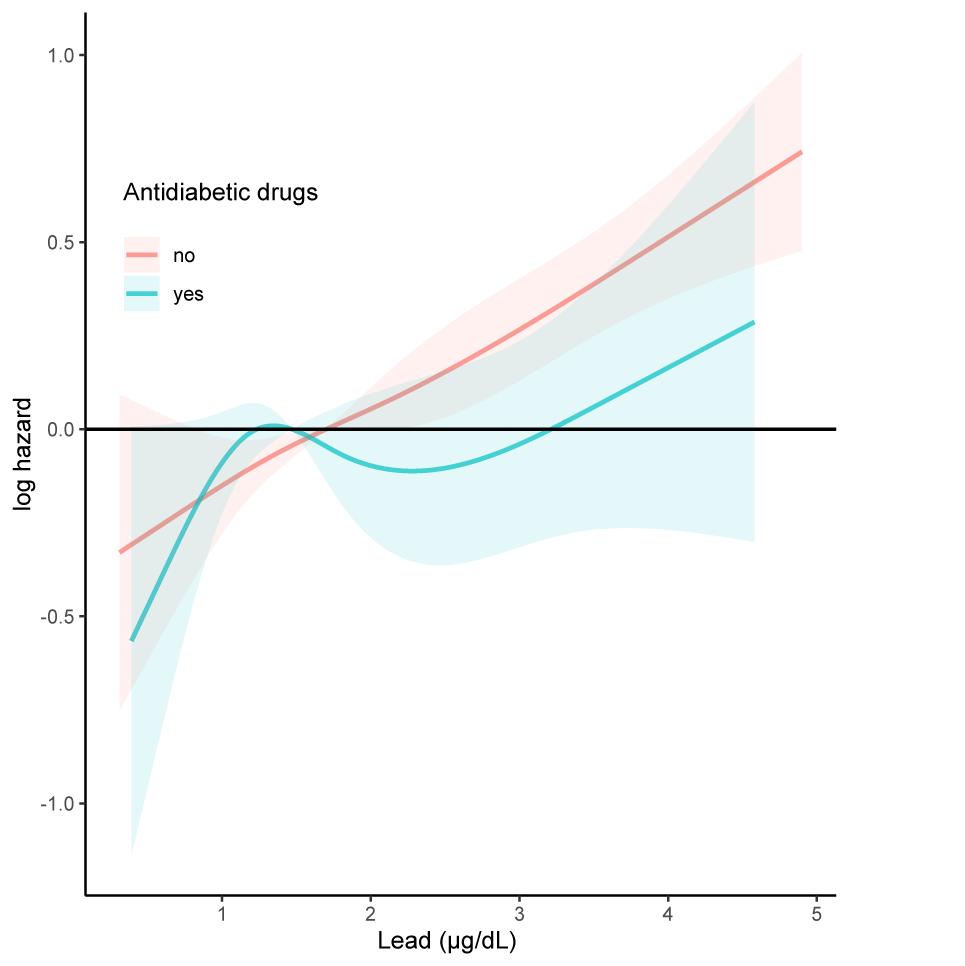

Supplement: Supplementary file 1 — Additional file 1: Supplementary Table 1. Thresholdeffect analysis of blood lead on all-cause mortality modified by age. SupplementaryTable 2. Threshold effectanalysis of blood lead on all-cause mortality modified by antidiabetic drugs. SupplementaryFigure 1. Adjustedcubic spline model of the association between hazard ratio of all-causemortality and blood lead levels in participants < 60 years old (P fornon-linear = 0.661) and ≥ 60 years old (P fornon-linear = 0.193). Supplementary Figure 2. Adjustedcubic spline model of the association between hazard ratio of all-causemortality and blood lead levels in participants without baseline antidiabeticdrugs (P for non-linear = 0.902) and with baseline antidiabetic drugs(P for non-linear = 0.196) [file 13690_2023_1148_MOESM1_ESM.zip › supplement_Figure2.tif]
