# Supplementary material for: Association of low-level lead exposure with all-cause and cardiovascular disease mortality in US adults with hypertension: evidence from the National Health and Nutrition Examination Survey 2003–2010
Source: Arch Public Health. 2023 Aug 14;81:146. doi: 10.1186/s13690-023-01148-6 (PMC10424362; doi:10.1186/s13690-023-01148-6)
Supplement: Supplementary file 1 — Additional file 1: Supplementary Table 1. Thresholdeffect analysis of blood lead on all-cause mortality modified by age. SupplementaryTable 2. Threshold effectanalysis of blood lead on all-cause mortality modified by antidiabetic drugs. SupplementaryFigure 1. Adjustedcubic spline model of the association between hazard ratio of all-causemortality and blood lead levels in participants < 60 years old (P fornon-linear = 0.661) and ≥ 60 years old (P fornon-linear = 0.193). Supplementary Figure 2. Adjustedcubic spline model of the association between hazard ratio of all-causemortality and blood lead levels in participants without baseline antidiabeticdrugs (P for non-linear = 0.902) and with baseline antidiabetic drugs(P for non-linear = 0.196) [file 13690_2023_1148_MOESM1_ESM.zip › Supplement_Table2.doc]

Supplementary Table 2 Threshold effect analysis of blood lead on all-cause mortality modified by antidiabetic drugs

|  | **Model 1 HR (95%CI) *P*** | **Model 2 HR (95%CI) *P*** | **Model 3 HR (95%CI) *P*** |
| --- | --- | --- | --- |
| **Antidiabetic agents** |  |  |  |
| Continuous | 1.40 ( 1.26,1.56) < 0.001 | 1.25 ( 1.11,1.41) < 0.001 | 1.09 ( 0.97,1.24) < 0.001 |
| Lead quartiles |  |  |  |
| Q1 | 1 | 1 | 1 |
| Q2 | 1.76 ( 1.15,2.68) 0.01 | 1.39 ( 0.92,2.10) 0.12 | 1.26 ( 0.86,1.86) 0.24 |
| Q3 | 1.94 ( 1.34,2.80) < 0.001 | 1.36 ( 0.94,1.97) 0.10 | 1.18 ( 0.83,1.67) 0.35 |
| Q4 | 2.54 ( 1.75,3.67) < 0.001 | 1.93 ( 1.33,2.79) < 0.001 | 1.42 ( 0.96,2.09) 0.08 |
| *p* for tend | 0.259 | 0.315 | 0.428 |
| **non-Antidiabetic agents** |  |  |  |
| Continuous | 1.53 ( 1.46,1.60 < 0.001 | 1.30 ( 1.23,1.37) < 0.001 | 1.25 ( 1.18,1.34) < 0.001 |
| Lead quartiles |  |  |  |
| Q1 | 1 | 1 | 1 |
| Q2 | 1.58 ( 1.22,2.03) < 0.001 | 1.15 ( 0.90,1.46) 0.26 | 1.24 ( 0.97,1.58) 0.08 |
| Q3 | 2.13 ( 1.68,2.69) < 0.001 | 1.28 ( 1.00,1.64) 0.05 | 1.33 ( 1.03,1.70) 0.03 |
| Q4 | 3.61 ( 2.92,4.47) < 0.001 | 1.93 ( 1.54,2.41) < 0.001 | 1.84 ( 1.45,2.33) < 0.001 |
| *p* for tend | < 0.001 | < 0.001 | < 0.001 |

HR, Hazard Ratio. CI, Confidence interval.

Model 1: Unadjusted model.

Model 2: Adjusted for age, sex and race.

Model 3: Confounders such as body mass index, education level, poverty income ratio, systolic blood pressure, diastolic blood pressure, smoker, drinker, estimated glomerular filtration rate, diabetes, hyperlipidemia, cancer, antihypertensive drugs, antihyperlipidemic agents, and antiplatelet agents were further adjusted on the basis of Model 2
